# Health and Safety in Temporary Work Zone Road Construction Project in Saudi Arabia: Risks and Solutions

**DOI:** 10.3390/ijerph191710627

**Published:** 2022-08-26

**Authors:** Saleh M. Alsultan, Fahad K. Alqahtani, Khalid F. Alkahtani

**Affiliations:** Department of Civil Engineering, College of Engineering, King Saud University, P.O. Box 800, Riyadh 11421, Saudi Arabia

**Keywords:** road safety, work zone, risk factors, crash data, traffic safety

## Abstract

Road project sites are dangerous and crash-prone, with many hazards that can cause injuries and can result in the deaths of road users or site-workers. Work zones for road construction or maintenance can potentially contribute to increasing these crashes. Many studies have addressed this issue; however, there is a lack of similar studies in Saudi Arabia. Therefore, this study contributes to developing safety practices for road work zones in Saudi Arabia by identifying, analyzing, and controlling the main risk factors. A survey approach was used to identify risk factors and potential countermeasures from road users’ and civil engineering experts’ perceptions. The main findings showed that most participants believed that the presence of work zones on the road might increase the probability of crash occurrence and that the highest risk factor that could cause a crash in a work zone is related to driver behavior. Both groups agreed that strict action against contractors or consultants who have safety violations would enhance road safety in work zones. Considering the findings of this study, decision-makers should take strong action to implement and improve road safety practices.

## 1. Introduction

The increasing demand for road construction, maintenance, or rehabilitation activities increases the number of work zones (WZs) on streets and highways. This is particularly hazardous and could cause significant safety concerns for road users and site-workers and affect the traffic flow. The presence of WZs for road construction, maintenance, or rehabilitation activities could potentially contribute to an increase in the number of traffic crashes or their severity. This has led to many negative impacts on society and the economy. Road crashes are a severe public health and development issue globally. The Global Status Report on Road Safety 2018 shows that traffic accidents lead to roughly 1.35 million lives lost around the world every year [1]. An additional 20 to 50 million people are injured yearly, with many sustaining permanent disabilities.

The Kingdom of Saudi Arabia (KSA) is considered one of the countries with the highest number of road traffic crashes. It recorded the highest number of deaths caused by road traffic crashes among the G20 countries in 2016. Additionally, the official statistics of Saudi Arabia indicate that there were 11,929,839 traffic crashes between 1970 and 2019, of which 208,949 were fatal [2]. These alarming numbers have resulted in more actions to curb traffic accidents and improve the quality of life through different initiatives, programs, and implementations. For example, after launching the Vision of KSA 2030 in 2016, the rate of deaths caused by road traffic crashes was reduced to about 18 per 100,000 people in 2018 compared to 27 per 100,000 people in 2016. The goal is to reduce these fatalities to less than 10 fatalities per 100,000 population by 2030. However, the implementation of these programs and actions appears to be inadequate for reducing the lives lost and the mitigation of safety costs arising from severe injuries, fatalities, legal obligations, and expenses. The rapid population and economic growth in Saudi Arabia have led to continuously expanding the road networks and enhancing the existing system. These road projects lead to an increased number of road work zones. However, the presence of road work zones leads to a rise in hazardous traffic conditions and traffic crashes and interrupts regular traffic flows.

Safety in road work zones (RWZs) remains a high-priority issue for roadway agencies mainly due to the limited understanding of the contributing factors of the crashes and the lack of perception of road works’ importance globally and locally. Accordingly, much research has been conducted to investigate the influences of many aspects of road work zone characteristics on RWZ safety [3,4,5,6,7,8,9,10,11,12,13,14,15,16,17,18,19,20,21,22,23,24,25,26,27,28,29,30]. The factors that contribute to crash frequency and/or severity include, but are not limited to, location, time and type of crash, roadway environment conditions and characteristics, and human factors. In detail, some studies agree that the presence of WZs increases the rate of crash occurrence [3,5,9,10,11]. For example, Akepati and Dissanayake (2011), Garber and Zhao (2002), and Salem et al. (2006) found that the work (i.e., activity) area is the most dangerous area and the predominant crash location [8,15,16].

Moreover, the majority of studies agree that the most common crash type is the rear-end crash, which is the most predominant crash in RWZs [4,5,8,15,16,27]. In terms of crash time frequency, the majority of studies revealed that most crashes occur during the daytime [4,17,19,24,27,29,30], but crashes that occur at night were found to be more severe [4,20,21,24,27,28,30].

Studies conducted by Li and Bai (2008) and Zhang and Hassan (2019) concluded that younger drivers had a higher probability of being involved in severe work zone crashes [22,24]. In addition, drivers older than 64 years and drivers aged between 35–44 had a higher possibility of causing fatal crashes [25]. Further, Chen and Tarko (2014) stated that the presence of a detour sign reduced crashes significantly [28].

In addition, previous studies have made many recommendations that could eliminate the risks of the road work zone and promote safety and mobility in the work zone, for example, the awareness of drivers about road work hazards and how this may affect their daily commute. Direct communication with motorists about precautions that should be taken when driving through WZs using social media campaigns, radio stations, effective educational programs, and seminars for the general public is recommended [17,21,24,29,30], as well as taking strict actions against those who commit safety violations and imposing heavy fines on speeding drivers [21,31]. In addition, using a cooperative intelligent transport system (C-ITS) for roadwork warnings would reduce bottlenecks at the roadworks’ entrance [32]. Regarding traffic control devices (TCDs), there is consensus that proper installation and maintenance of related TCDs and strictly maintaining the condition and reflectivity of those devices will improve safety in WZs [9,17,18,21,24,31]. It is clear that the majority of the studies reported earlier were not conducted locally, which implies the need for a local study in the area of research since the safety of WZs is highly influenced by differences in the area, driver knowledge, awareness, type of WZ, traffic conditions and density, WZ duration, and the reliability of the crash data. Moreover, to the authors’ knowledge, there is still no research paper that assesses the potential risk factors of the road work zone and corresponding suggestions to mitigate those risks based on road users’ and civil engineering experts’ opinions and explores the differences between the two groups in assessing those potential risk factors and suggestions.

These factors are highly sensitive to the study region, and there is a lack of understanding of the magnitude of the crashes’ causes and their consequences on Saudi Arabia’s road projects. Therefore, this study aims to improve road projects’ health and safety practices in Saudi Arabia by identifying and analyzing the main risk factors that cause crashes in RWZs and addressing the potential suggestions and recommendations to minimize these risks. This study could be used as a guide for successful construction safety program implementation in road projects as well as safety policy development in the Saudi construction industry.

## 2. Methodology

### 2.1. Study and Survey Design

In order to achieve the goals of this study, a survey approach that addresses RWZs’ potential sources of risk and the suggested solutions, including the findings from previous similar research, was used. This survey was designed to cover both road users and experts, represented by professionals from different cities, to increase the accuracy and reliability of the results. This is important to understand the safety perception of road users toward WZs. The safety perception affects road user decisions yet is not easily understood by looking at crash data [33]. Civil engineers represent the main project parties, in addition to owners, contractors, and consultants who have knowledge and experience in transportation and traffic engineering.

**Figure 1:**
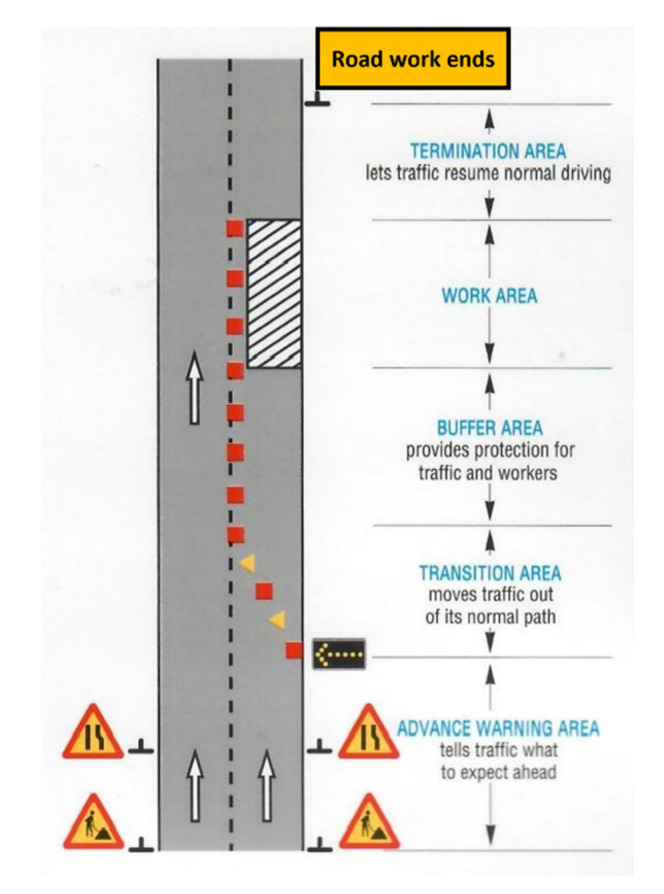
Areas of road work zone.

The survey consists of three parts addressed to both groups. The first part of the survey consists of several questions related to demographic data (i.e., socio-economic information). The second section focuses on crash causes in RWZs and measures drivers’ risk perception. The second part of the survey is divided into two subsections. The first subsection contains six general questions regarding the respondent’s evaluation of the influences of the presence of RWZs, number of existing RWZs, area of RWZs as shown in Figure 1, and warning signs and their effect on crash occurrence probabilities. It also asks the number of times participants were involved in a traffic crash generally, and particularly in RWZs. The other subsection consists of 17 questions that address the potential risk factors of RWZs. The participants rate the factors using a Likert scale from 1 to 10 (where 1 is not probable at all, and 10 is very probable). The last part of the survey comprises 14 suggestions for improving road safety in RWZs. Participants rate each suggestion using a Likert scale from 1 to 10 (where 1 is not probable at all, and 10 is very probable) based on their opinions.

The sample size of the study was obtained using the formula given below (Equation (1)). Using this formula, the sample size of the current study was 225, assuming that the population size of the sample is vehicles registered in Saudi Arabia until the end of 2019. The number of vehicles registered until 2019 was approximately 12 million [34]. A web survey was used to reach the targeted audience.

(1) $$Sample\;size=\frac{\frac{z^{2}Xp\left(1-p\right)}{e^{2}}}{1+\frac{z^{2}Xp\left(1-p\right)}{e^{2}N}}$$

where, N = Population Size = 12,000,000

Confidence Level (%) = 90

*e* = Margin of Error (%) = 5.5

*z* = z-score = 1.65

*p* = Sample proportion (50%).

### 2.2. Preliminary Analysis of the Survey

Before analyzing the data, a survey data cleaning method was implemented to obtain the most accurate survey data after collecting the survey responses. Survey data cleaning involves identifying and removing responses from individuals who did not answer the questions thoughtfully using three criteria or filters. The first filter excluded those who did not complete the whole survey. The second filter excluded respondents who blindly selected the same answer/response for each question throughout the entire survey (e.g., choose the first option for each question). Finally, the third filter excluded inconsistent responses (e.g., when choosing his highest education level as high school and his occupation as a civil engineer). After defining and cleaning the data, they were inspected, and the nature of the variables was explored. One-way and two-way contingency table analyses were performed. The former approach is fundamental analysis, and it helps to obtain the basic information about the survey participants and crash causes data. The latter approach was performed to examine the association between the observed variables and identify any specific data trends; it is commonly used to deal with survey questions.

The appropriate statistical analyses were used according to the data type from a question. Descriptive statistics such as frequencies, percentages, averages, and standard deviations were used to present an overview of findings from the study population. Additionally, inferential statistics were used to explore the statistically significant differences among groups and the association between variables, for example, the chi-square test and the Independent-samples T-test. It is worth mentioning that because of violating assumptions of the Pearson chi-square, a likelihood-ratio chi-square was selected to determine the association between the variables.

The data were analyzed using Statistical Package for Social Science (IBM SPSS version 25, Armonk, NY, USA).

## 3. Results and Discussion

### 3.1. Distributions of The Survey Sample

Prior to distributing the survey, a pilot study was conducted to check its reliability. Reliability analysis was calculated using Cronbach’s alpha coefficient. Cronbach’s alpha coefficients for all parts of the survey ranged from (0.878–0.948), which is acceptable and refers to the high reliability of the study tool.

A total of 410 responses were received before the data cleaning process. In the end, 223 road users’ and 98 civil engineers’ responses were used in this study. The percentage completion rate of the survey was 78%. Table 1 summarizes the frequencies and percentages of the road users’ and civil engineers’ data to gain more understanding of the distribution of the targeted sample. It can be seen that the ages of most participants ranged from 25 to 44 years. More than 80% of the road users’ participants have a bachelor’s degree or higher. Most of the participants have valid driving licenses. The civil engineers’ participants represent different organizations, 44.9% owners, 25.5% consultants, and 29.6% contractors. This means the opinions of civil engineers represent all sectors. Most participants (80%) have more than 10 years of driving experience (driving years) which means that the study participants have substantial knowledge and understanding of driving issues; therefore, we have obtained reliable and accurate data through the structured survey.

Table 2 summarizes the frequencies and percentages of both groups regarding the crashes’ causes. Almost all respondents find RWZs in their daily trips (i.e., 99% and 100% for road users and civil engineers, respectively). Therefore, there is a need to consider the safety of RWZs. Additionally, their opinion about road safety issues within WZs could be valuable. Most participants reported that they believe that WZs on the road may increase the probability of crash occurrence by 83% and 76.5%, respectively, for road users and civil engineers. The difference between the two groups might be because civil engineers trust their work more. Most of the opinions corresponded with most studies’ findings that state that WZs increase the rate of crash occurrence [3,5,9,10,11]. Specifically, 55.2% of road users and 60.2% of civil engineers said that the transition area was the area with the highest risk probability of causing a crash in the WZ, followed by the advance warning area, 18.4% of road users, and 21.4% of civil engineers. This agrees with studies conducted by Jin and Saito (2009) and Khalil and Samir (2018) that stated the most crash-prone area location is the transition area [17,18]. In addition, approximately 92% of both groups reported that they obey WZ ahead signs by reducing their vehicle speed. Respondents who had a traffic crash at least once or more were 73.5% of road users and 65.3% of civil engineers. Finally, 29 road users (13%) and 8 civil engineers (8.16%) were involved in a traffic crash in RWZs.

### 3.2. Exploring Relationships between Variables

A chi-square test for independence was used to test hypotheses that answer the study question of whether there is a relationship between two categorical variables. It was performed for the two groups separately. The test was performed between the observed six general questions (listed in the previous section). It covers the number of times the participants were involved in a traffic crash generally, or particularly in an RWZ, with the socio-economic information to determine the association at a significance level of 0.05. Table 3 presents the chi-square test outputs that had a statistically significant association between variables and factors for the road users’ and civil engineers’ data.

Regarding the road users’ data, the results show that none of the road users who had comprehensive vehicle insurance disagreed with the statement that the presence of a WZ on the road would increase the probability of crash occurrence. Additionally, 84.1% of those with third-party insurance agreed with the statement. This safety perception of road users toward WZs should be taken into consideration by each entity to improve safety in WZs. Further, the majority of the road user participants, regardless of their education levels, agreed that the transition area is the riskiest area that could cause a crash in WZs. Thus, there must be more precautions in the transition area. Concerning involvement in a traffic crash, the office public employees had the highest number of crashes among other occupations.

For the civil engineers’ group, 83% of the participants who have driving experience of more than 10 years believed that the presence of a WZ on the road would increase the probability of crash occurrence. In contrast, 33.3% of them with driving experience between 1–3 years believed that the presence of a WZ on the road would not increase the probability of crash occurrence. This indicates that those with more years of driving experience perceive that the WZ is risky. In addition, none of the civil engineers with postgraduate degrees had been involved in RWZ-related crashes.

### 3.3. Risk Factors Assessment and Identification

After reviewing the previous research related to the safety of RWZs, there were 17 potential risk factors identified to be asked of the respondents in order to evaluate them. These risk factors generally represent three categories, driver behavior, WZ setup, and worksite management. It is crucial to assess those factors to identify potential hazards that could face the drivers and relate them to the road work zone.

In order to check if there was a statistically significant difference in the mean scores of risk factors to the RWZ for the two groups regarding their opinion on potential risk factors, an independent-samples *t*-test has been employed. Thus, for risk factors that had significant differences in mean and values less than *p* < 0.05, the null hypothesis would be rejected and conclude that the mean of the four risk factors for road users and civil engineers are significantly different for some risk factors. There were only four risk factors that had a statistically significant difference in the mean, namely, a poor design of the detour, a large speed variance between the posted temporary speed limit and permanent speed limit within short distances, no longitudinal and transverse grades for rain drainage in the RWZ or the detour, and drivers not responding to temporary warning signs in RWZs to reduce vehicle speed. However, that could assess the importance of the finding by calculating the effect size. Based on these results, Eta squared was employed to estimate the size effect of the difference. Eta squared effect size statistics indicate the proportion of variance of the dependent variable explained by the independent variable, and its values range from 0 to 1. The interpretation guideline of values is 0.01 = small effect, 0.06 = moderate effect, and 0.14 = large effect, as proposed by Cohen (1988) [35]. The effect size indicates the magnitude of the differences between road users’ and civil engineers’ opinions. The magnitude of the differences in the means of risk factors was very small for the same factors (0.0175, 0.0182, 0.0134, and 0.0138). Therefore, because of the small rate differences, both groups are combined to obtain the mean of risk factors that represent both opinions.

That gives an indicator of which most dangerous risk factor has the highest probability of causing a crash in RWZs. Hence, the responsible agency or decision-makers should take urgent action to resolve the issue and improve road safety on the site. By sorted risk factors, the highest factor’s mean indicates the most probable risk factor that could cause a crash in the RWZ, as well as other factors sequentially ordered based on the most probable risk factor to the lowest probable that could cause a crash on the RWZ. Table 4 shows the mean of each group’s opinion and the combined mean that represents both groups’ opinions.

The results show that both groups agreed that the highest risk factor is reckless or aggressive driving through the WZ. This implies the importance of accommodating human behavior when designing RWZs. This finding agrees with the findings of Akepati and Dissanayake (2011), which stated that the primary contributing factors in RWZ crashes were inattentive driving and following too close [15]. In addition, Zhang and Hassan (2019) mentioned that most crashes in WZs are related to human error [22]. Additionally, some studies’ findings regarding driver behavior and human error revealed that the most frequently contributing factors that cause WZ-related crashes are driver inattention and tailgating [4,23,24,25]. Additionally, driving behavior was found by Bharadwaj et al. (2019) as the most critical risk factor in a WZ, and particularly inattention has the highest level of risks among all types of driver behavior [23]. This was followed by risk factors related to setting the RWZ, such as lack of lighting in RWZs, lack of temporary road signs, and poor detour design. Thus, safety professionals should not rely only on the related manuals or guidelines to set the RWZ, such as the Manual on Uniform Traffic Control Devices (MUTCD) but also should continue to monitor for crashes and other measures of safety to make sure that the RWZ is substantively safe. However, they agreed that the presence of workers on the site seems less likely to be a risk that could cause a crash. The rest of the sorted risk factors could give indicators of which risk factor at the site must be resolved urgently by the responsible agency or safety professional.

### 3.4. Suggestions to Improve Road Safety in Road Work Zone

The last part of the survey contained suggestions to improve road safety in WZs. Based on previous literature, many researchers have proposed various suggestions and recommendations that they believe will enhance safety and mobility in work zones. Some of those suggestions and recommendations were selected, new suggestions were added, and the respondents were asked to evaluate them. This will help decision-makers to choose the most effective suggestions to take corrective action to eliminate the risk factors.

The results show that five suggestions that had a significant difference in the mean scores between the two groups were (1) awareness among drivers about road work hazards through social media campaigns, (2) stronger collaboration between government agencies in terms of improving road safety, (3) better and updated work zone standards that reflect local conditions, (4) provide a database system for documenting and reporting the crashes causes, and (5) having a certificated safety engineer or professional on the project for road risk assessment. The magnitude of rate differences of suggestions between road users and civil engineers are small (0.0129, 0.0163, 0.0187, 0.0144, and 0.0277). Because of the small differences, both groups’ opinions were combined to get the overall mean of suggestions representing both opinions. By sorted suggestions, the highest suggestion’s mean indicates the most probable suggestion that will enhance the road works’ health and safety practices and could lead to minimizing road crashes. Sequentially mean ordered based on the most probable suggestion to lowest probable are shown in Table 5. The results show that both groups overall recommended that the most effective action that could be taken to enhance WZ safety is strict action against contractors or consultants who commit safety violations. This suggestion could indicate that the current status of RWZs is unsafe due to contractors’ or consultants’ work through WZ setup and worksite management. Moreover, this suggestion is consistent with many researcher recommendations [3,11,31]. The authors believed that involving some contracting strategies such as incentives and disincentives within contracts to shorten the WZ duration can be considered as a type of safety benefit. The second overall ranked suggestion was a stronger collaboration between government agencies in terms of improving road safety. This is in line with a previous study conducted by Ullman et al. (2008), stating that one of the effective management policies, procedures, and practices that would enhance the safety of the work zone is coordinating between multiple agencies [31]. The authors believed this kind of coordination has a potential safety benefit and would decrease the frequency and significance of traffic congestion. Although the suggestion of having a certified safety engineer or professional on the project for road risk assessment was ranked third by both groups, it was ranked highest by the civil engineers. Therefore, it is recommended to conduct a road safety audit (RSA) of road work schemes where the new road project is improving (or connected to) a highway already in operation to assess the proposed temporary traffic management design plans and to inspect during daylight and at night for all likely traffic movements. Keep in mind that the main goal is to guide all road users past the WZ safely and efficiently while protecting the workers in the zone. Interestingly, there was an awareness of the importance of having updated standards and a better database system and analysis as both agreed overall on the fourth and fifth ranks, respectively. Most of the researchers in the literature mentioned that when the WZ crashes data are misclassified, that will lead to a lack of detailed data [5,17,30,31,36]. Additionally, they suggested the development of uniform standards for reporting WZ crashes and shared data to better facilitate work zone crashes investigation. Thus, it is vital to have such systems for collecting and reporting WZ-related crash data from related agencies and providing local officials and interested groups with work zone safety-related information. Additionally, most respondents thought that the presence of better and good condition TCD on WZ sites would definitely improve the safety of the WZs. That agrees with studies by Garber and Woo (1990), Jin and Saito (2009), Khalil and Samir (2018), Li and Bai (2008), Osman et al. (2018), and Ullman et al. (2008), which emphasized that the presence of a good condition of the TCD is effective and has a positive impact on work zone safety [9,17,18,21,24,31]. In terms of using developed technologies, it seems that deploying smart technologies in smartphone applications or within vehicles would affect and improve the health and safety of road work zones.

Lastly, as shown below in the list, the lowest suggestion is to consult with road users on matters affecting their health and safety in RWZs and get their feedback. This could mean the risk factors were apparent and only needed the decision-makers to take corrective action to eliminate them.

These suggestions can be described as those that the road users and civil engineers perceive to need more focus and apply by decision-makers to improve road health and safety practices.

Applying those suggestions would help reduce traffic issues in the work zone area and make the traffic flow smoothly without disruption. Additionally, it will reduce the loss of road users’ and workers’ lives and mitigate safety costs arising from severe injuries, fatalities, legal obligations, and expenses. There is consensus that the effects of these countermeasures have positive impacts on WZ safety.

## 4. Conclusions

Saudi Arabia has many kilometers of roads due to its vast area. In order to achieve the Vision of KSA 2030 of reducing the number of fatalities due to traffic crashes and improving quality of life, it is vital to investigate the health and safety of road project sites during construction and maintenance. The presence of WZs on the road raises numerous risk factors. Moreover, those risk factors could potentially contribute to increasing crashes in WZs. The main objectives of this study were to identify the potential risk factors and the corresponding suggestions to mitigate those risks. Detailed literature was reviewed to get a better understanding of similar conditions worldwide. Additionally, a multiple survey approach was carried out to obtain data from road users and civil engineering experts. The survey addresses RWZ issues of potential factors and corresponding suggestions to mitigate those risks. Lastly, one-way analysis and two-way contingency table analysis were applied.

The results show that most road users and civil engineers find RWZs in their daily trips. Most participants believed that the WZs on the road might increase the probability of crash occurrence. Therefore, there is an issue facing drivers every day while driving. The majority of the participants believed that the transition area is the highest risk area in an RWZ. Regarding exploring associations, relationships were found between some variables with factors related to socio-economic information.

In addition, several risk factors with respect to driver behavior, WZ setup, and worksite management were identified and ranked. Similarly, several suggestions were identified to reduce the risk in WZs.

Both groups agreed that the most contributing factors for crashes are:

- Reckless or aggressive driving through the work zones;
- Lack of lighting in RWZs;
- Lack of temporary road signs in RWZs.

The probable suggestions that would improve road safety in the WZs based on the participants’ opinions are:

- Strict action against contractors or consultants who commit safety violations;
- Stronger collaboration between government agencies in terms of improving road safety;
- Have a certificated safety engineer or professional on the project for road risk assessment.

The main findings of this study addressed the main potential risk factors of RWZs that could cause crashes in RWZs. Additionally, it suggested appropriate actions for decision-makers that will enhance the road works safety practices and could lead to minimizing road risks. It is highly recommended to consider these findings, including providing a database system for documenting and reporting the crashes caused by government agencies for more efficient management and analysis in the future. Based on the research outcomes, the authors suggested focusing on intelligent transport systems (ITSs) to be considered in future studies, such as testing and measuring how ITSs technologies could affect driver behavior in the road work zone. Additionally, it could quantify the risk factors and the suggestions by using a regression model to describe the relationship between them and, locally, study the overlapping work between the government agencies in terms of traffic safety in Saudia Arabia and the reasons for the reckless and aggressive driving behavior.

## Figures and Tables

**Table 1:** Distribution of road users and civil engineers survey sample.

Variable	Categories	Number of Participants		Percentage of Participants (%)	
		Road Users	Civil Engineers	Road Users	Civil Engineers
Age	17–19 years	2	0	0.9	0
	20–24 years	12	2	5.4	2
	25–29 years	32	25	14.3	25.5
	30–34 years	47	22	21.1	22.4
	35–39 years	41	19	18.4	19.4
	40–44 years	33	16	14.8	16.3
	45–49 years	29	3	13	3.1
	50–54 years	14	3	6.3	3.1
	55–59 years	7	3	3.1	3.1
	60–64 years	6	4	2.7	4.1
	older than 64 years	0	1	0	1
Highest education level	Postgraduate degree	68	21	30.5	21.4
	Bachelor’s degree	112	77	50.2	78.6
	Diploma	19	0	8.5	0
	High school	22	0	9.9	0
	Intermediate	2	0	0.9	0
Driving license status	Valid	203	96	91	98
	Expired	11	2	4.9	2
	I don’t have	9	0	4	0
Driving experience (driving years)	Within 1 year	14	0	6.3	0
	1–3 years	2	6	0.9	6.1
	4–6 years	10	4	4.5	4.1
	7–9 years	17	10	7.6	10.2
	≥10 years	180	78	80.7	79.6
Vehicle’s insurance type	Third-party	158	63	70.9	64.3
	Comprehensive	45	22	20.2	22.4
	Expired	20	13	9	13.3
Occupation	Office public employee	142		63.68	
	Office private employee	58		26	
	Unemployed	14		6.28	
	University student	8		3.59	
	School student	1		0.45	
	Civil engineer	0	98	0	100
Role of civil engineers’ organizations	Owner		44		44.9
	Contractor		29		29.6
	Consultant		25		25.5

**Table 2:** Responses of the crashes’ causes part (six general questions).

Variable	Categories	Number of Participants		Percentage of Participants (%)	
		Road Users	Civil Engineers	Road Users	Civil Engineers
The presence of work zones on the road would increase the probability of crashes	Yes	185	75	83	76.5
	No	15	14	6.7	14.3
	Unsure	23	9	10.3	9.2
Frequency of road work zone in your daily trips	Never	2	0	0.9	0
	Rarely (once)	37	15	16.6	15.3
	Sometimes (Twice)	77	31	34.5	31.6
	Usually (3–5)	75	43	33.6	43.9
	Always (+6)	32	9	14.3	9.2
Opinion of which area has the highest risk probability that could cause a crash on WZ	Before advance warning area	28	7	12.6	7.1
	Advance warning area	41	21	18.4	21.4
	Transition area	123	59	55.2	60.2
	Buffer area	13	5	5.8	5.1
	Work area	15	5	6.7	5.1
	Termination area	3	1	1.3	1
Obligation to work zone signs	Yes	205	91	91.9	92.9
	No	18	7	8.1	7.1
Involvement in a traffic crash	Never	59	34	26.5	34.7
	Once	41	14	18.4	14.3
	Twice	54	32	24.2	32.7
	3–5 times	58	16	26	16.3
	More than 5	11	2	4.9	2
Involvement in a traffic crash in road work zone	Yes	29	8	13	8.16
	No	194	90	87	91.84

**Table 3:** Association between the observed variables.

		Road Users			Civil Engineers		
Variable	Factor	Chi-Square Value	df	*p* Value	Chi-Square Value	df	*p* Value
The presence of work zones on the road would increase the probability of crashes occurrence	Driving experience			Insignificant	16.061	6	0.013 *
	Vehicle’s insurance type	10.999	4	0.027 *			Insignificant
Opinion of which area has the highest risk probability that could cause a crash on WZ	Highest education level	34.681	20	0.022 *			Insignificant
Involvement in a traffic crash	Occupation	9.804	4	0.044 *			Insignificant
Involvement in a traffic crash in road work zone	Highest education level			Insignificant	4.049	1	0.044 *

* Significant at α = 0.05.

**Table 4:** The mean of each group and the combined mean of the risk factors.

No.	Risk Factors	Mean		
		Road Users (N = 223)	Civil Engineers (N = 98)	Both (N = 321)
1	Reckless or aggressive driving	8.63	8.92	8.72
2	Lack of lighting in road work zones	8.50	8.92	8.63
3	Lack of temporary road signs (warning signs, work zone signs, regulatory signs, informative signs, etc.)	8.14	8.11	8.13
4	Poor design of the detour	7.72	8.41	7.93
5	Drivers not responding to temporary warning signs in road work zones by starting to reduce vehicle speed once they are warned	7.65	8.19	7.82
6	Drivers unaware of road works risks	7.49	7.89	7.61
7	Not installing the concrete barriers appropriately	7.62	7,26	7.51
8	Absence of new road line markings	7.25	7.61	7.36
9	Road surface status (i.e., paved or not paved, cracks and holes)	7.38	7.29	7.36
10	Worksite vehicles entering or exiting the road work zone	7.16	7.61	7.30
11	Not removing temporary road signs, cones, or road humps after work had been done	7.29	7.27	7.28
12	Unclean road surface and worksite due to works (i.e., sand, debris, or oil)	7.36	6.97	7.24
13	Lack of manual flaggers	6.83	6.66	6.78
14	The high speed variance between posted temporary speed limit and permanent speed limit within short distances	6.42	7.18	6.65
15	No longitudinal and transverse grades for rain drainage in the road work zone or in the detour	6.46	5.80	6.26
16	No pedestrian crossing lines (zebra crossing lines)	6.28	6.08	6.22
17	Presence of workers on the site	5.09	5.11	5.10

**Table 5:** Suggestions to improve road safety in the work zone.

No.	Suggestions	Mean		
		Road Users (N = 223)	Civil Engineers (N = 98)	Both (N = 321)
1	Strict action against contractors or consultants who commit safety violations	8.41	8.76	8.52
2	Stronger collaboration between government agencies in terms of improving road safety	8.23	8.77	8.38
3	Having a certificated safety engineer or professional on the project for road risk assessment	8.08	8.85	8.31
4	Better and updated work zone standards that reflect local conditions	7.96	8.56	8.14
5	Provide a database system for documenting and reporting the cause of crashes by government agencies for more efficient management and analysis	7.91	8.5	8.09
6	Better Traffic Control Devices (TCD) (e.g., signs, channelizing devices, and arrow panels)	7.83	8.23	7.96
7	Using developed technologies, for example, speed radar, cameras, or linking and updating the road status within smartphone applications or within vehicle navigation could lead to minimizing road risks	7.86	8.17	7.95
8	Informing road users about work zones before starting the work	7.7	7.99	7.79
9	Daily road work zone inspections by a project manager	7.53	8.02	7.68
10	Awareness of the drivers about road works’ hazards through social media campaigns	7.44	8.01	7.61
11	Increasing the penalties for speeding or committing other traffic violations while in a construction work zone	7.36	7.74	7.48
12	Safety tools equipped on a car, such as anti-lock brakes (ABS), lane departure warning, traction control, active rollover protection (ARP), automatic braking system, blind-spot detection, etc., could prevent accidents	7.34	7.57	7.41
13	Display information about the reason, time of completion, or length of ongoing road work	7.07	6.84	7.00
14	Consult with road users (feedback) on matters affecting their health and safety in road work zones	6.9	6.66	6.83

## Data Availability

Not applicable.

## Abbreviations

WZ	Work Zone
RWZ	Road Work Zone
KSA	Kingdom of Saudi Arabia
G20	Group of Twenty (20 countries)
TCD	Traffic Control Device
MUTCD	Manual on Uniform Traffic Control Devices
C-ITS	Cooperative Intelligent Transport System
RSA	Road Safety Audit
ITS	Intelligent Transport System
